# Predictors of Mortality and Long-Term Outcome in Patients with Anterior STEMI: Results from a Single Center Study

**DOI:** 10.3390/jcm10235634

**Published:** 2021-11-29

**Authors:** Giulia Ferrante, Lucia Barbieri, Carlo Sponzilli, Stefano Lucreziotti, Diego Salerno Uriarte, Marco Centola, Monica Verdoia, Stefano Carugo

**Affiliations:** 1 Division of Cardiology, ASST Santi Paolo e Carlo, 20142 Milan, Italy; giulia.ferrante@asst-santipaolocarlo.it (G.F.); carlo.sponzilli@asst-santipaolocarlo.it (C.S.); stefano.lucreziotti@asst-santipaolocarlo.it (S.L.); diego.salerno@asst-santipaolocarlo.it (D.S.U.); marco.centola@asst-santipaolocarlo.it (M.C.); 2 UOC Cardiology, Fondazione IRCCS Cà Granda Ospedale Maggiore Policlinico, 20122 Milan, Italy; stefano.carugo@unimi.it; 3 Division of Cardiology, Nuovo Ospedale degli Infermi, ASL Biella, 13875 Biella, Italy; monica.verdoia@gmail.com

**Keywords:** STEMI, multivessel disease, cardiogenic shock, complete revascularization

## Abstract

Anterior ST segment elevation myocardial infarction (A-STEMI) has the worst prognosis among all infarct sites due to larger infarct size and the higher cardiac enzyme release. We retrospectively analyzed 584 A-STEMI undergoing urgent coronary angiography from October 2008 to April 2019. The median follow-up time was 1774 days with a minimum of a 1-year follow-up for 498 patients. In-hospital mortality was 8.6%, while long-term, all-cause mortality and 1-year mortality were 18.8% and 6.8%, respectively. The main predictors for in-hospital mortality were ejection fraction (LV-EF), baseline estimated glomerular filtration rate (eGFR), female gender and cardiogenic shock (CS) at admission, while long-term predictors of mortality were age, coronary artery disease (CAD) extension and LV-EF. Patients presenting with CS (6.5%) showed a higher mortality rate (in-hospital 68.4%, long term 41.7%). Among 245 patients (42%) with multivessel disease (MVD), complete revascularization (CR) during the index procedure was performed in 42.8% of patients and more often in patients with CS at admission (19.1% vs. 6.1%, *p* = 0.008). Short- and long-term mortality were not significantly influenced by the revascularization strategy (CR/culprit only). Our study confirmed the extreme fragility of A-STEMI patients, especially in case of CS at admission. LV-EF is a powerful predictor of a poor outcome. In MVD, CR during p-PCI did not show any advantage for either long- or short-term mortality compared to the culprit-only strategy.

## 1. Introduction

Anterior STEMI (A-STEMI) has the worst prognosis among all infarct sites mostly due to larger infarct size and to the higher cardiac enzyme release. Patients with A-STEMI experience a more complicated in-hospital and follow-up course and are at greater risk for acute regional dilatation and thinning of the infarct zone. An accurate prognostic assessment is therefore mandatory, and the improvement in risk stratification of acute myocardial infarction (AMI) patients is an important contributor to better and more efficient patient management. Approximately 40% to 65% of patients presenting with STEMI had multivessel coronary artery disease (MVD) with concomitant obstructive non infart-related artery (IRA) stenosis [1,2]. While it is recommended to always treat the IRA, it remains unresolved whether complete revascularization (CR), either immediate or staged, should be undertaken in the setting of stable STEMI, with discordant evidence about its clinical effect on outcome [2,3,4]. Previous studies addressing the management of non-IRA lesions produced conflicting results and, in last decades, several clinical trials have been performed in order to further investigate the best and safety approach in this setting [5,6,7,8,9,10,11,12]. A particular scenario in which timing of myocardial revascularization plays a central role is represented by cardiogenic shock (CS), a serious and often fatal complication of A-STEMI, whose incidence ranges from 4% to 15%. Despite the advances in pharmacological treatment and device technology over the last decades, leading to a steady reduction in mortality, CS remains the main cause of death, with hospital mortality rates still approaching 50% [13]. To date, several studies have assessed revascularization modality and timing among patients with CS with conflicting results [14,15,16,17]. On the basis of these assumptions, the aim of the present study was to evaluate the main predictors of short- and long-term mortality in patients with A-STEMI focusing in particular on high-risk subgroups such as patients presenting with CS or MVD.

## 2. Materials and Methods

From a total of 1296 consecutive STEMI patients undergoing urgent coronary angiography and/or percutaneous coronary intervention (PCI) from October 2008 to April 2019, we selected and retrospectively analyzed those presenting with A-STEMI (*n* = 584). Patients were treated with optimal medical therapy according to current good clinical practice. Coronary angiography was urgently performed through the femoral or radial access at the discretion of the interventional cardiologist. Critical stenosis was defined by vessels ≥1.5 mm in diameter with >70% stenosis. MVD was defined as >70% stenosis in at least 2 major vessels (≥2 mm). Left ventricular ejection fraction (LV-EF) was calculated by standard trans-thoracic echocardiography before discharge and, for those patients who died during hospitalization, only the urgent LV function assessment in the emergency department was available. Estimated glomerular filtration rate (eGFR) was calculated using the CKD-EPI formula [18]. Anemia was defined as a hemoglobin level of less than 13 g/dL in men and less than 12 g/dL in women. We collected follow-up information at clinic visits or during phone calls. Data from the “Registro Regionale Lombardia” were also used for accurate long-term mortality evaluation. In-hospital, 1-year and long-term follow-up mortality and their main predictors were evaluated for the entire population. A sub analysis among patients with MVD was then performed in order to evaluate the potential impact of CR on short and long term mortality. Our study was undertaken in accordance with the Declaration of Helsinki. All data were collected in a dedicated database protected by password.

### Statistical Analysis

Statistical analysis was performed using the SPSS 23.0 statistical package. Continuous variables are expressed as mean + standard deviation and categorical data as percentages. Analysis of variance and the chi-square test were used for continuous and categorical variables, respectively. Cox proportional hazards regression and logistic regression modelling were used to identify independent clinical and procedural predictors associated with short- and long-term mortality. Variables included in the models were represented by main known risk factors for mortality in STEMI patients (age, Killip class and CS at admission, ACC, intraprocedural IABP, anemia, female gender, eGFR, hypertension, diabetes, LV-EF and CAD extension). Survival curves were calculated by Kaplan–Meier method.

## 3. Results

Our population is represented by 584 consecutive patients undergoing coronary an-giography and/or PCI for A-STEMI. The median follow-up time was 1774 days (IQR 2052 days) with a minimum of 1-year follow-up for 498 patients. The baseline clinical and procedural characteristics of the whole population and divided in two groups according to survival are listed in Table 1.

**Figure 1:**
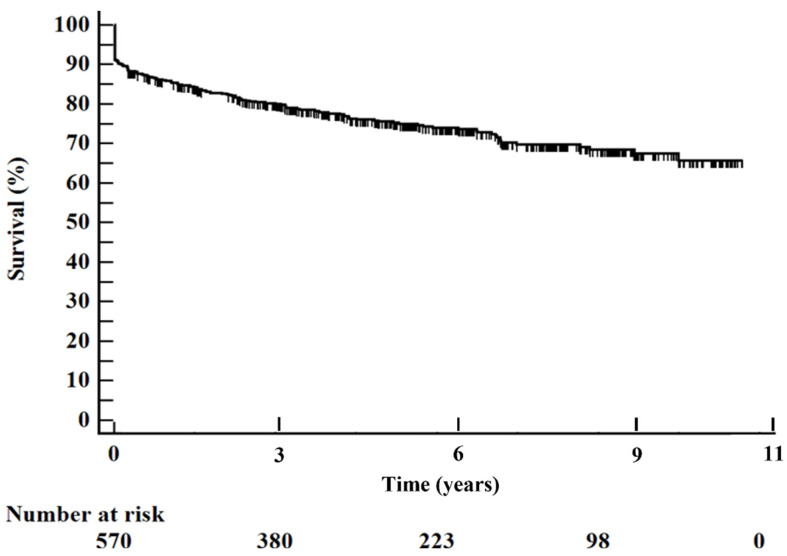
Overall survival at maximum follow-up in the total cohort of patients.

Patients were more often males (70.7%) with a mean age of 66.2 +/− 13.6 years. At admission 5% showed Killip class 3, 6.5% presented with CS, and cardiac arrest was the first manifestation for the 3.3%. In-hospital mortality was 8.6%, while long-term all-cause mortality and 1-year mortality were 18.8% and 6.8%, respectively. The Kaplan–Meier curve represents the survival of the entire population from the hospital admission to the maximum follow-up time (Figure 1).

As previously reported in Table 1, non-surviving patients (*n* = 161) were older (77.5 +/− 10.8 vs. 62.5 +/− 12.2 years old), female (43.2% vs. 34.4%), anemic (26.4% vs. 18.2%) and had an impaired renal function at the baseline (eGFR ≤ 30 mL/min/m^2^ in 13.3% vs. 2%). Moreover, a higher Killip class (≥3 in 32.4% vs. 4.3%) and cardiogenic shock (20.9% vs. 1.7%) at admission were more often represented. At coronary angiography three vessel disease (26.3% vs. 11.6%), left main involvement (10.5% vs. 0.2%) and the need for IABP support (13.5% vs. 1.9%) with the more frequent femoral approach (74.3% vs. 52.6%) in performing PCI was observed. These results were confirmed using Cox regression analysis, where the main predictors for long-term mortality were age (HR 1.12, *p* < 0.001), CAD extension (HR 1.41, *p* = 0.003) and LV-EF (HR 0.96, *p* < 0.001). At the 1-year follow up only age (OR 1.15, *p* = 0.003) and Killip class at admission ≥ 3 (OR 4.17, *p* = 0.04) were identified as independent predictors for mortality. Focusing on in-hospital mortality, LV-EF (OR 0.93, *p* = 0.017), baseline eGFR (OR 0.97, *p* = 0.03), female gender (OR 9.06, *p* = 0.01) and CS at admission (OR 29.56, *p* < 0.001) were the main significant predictors (Table 2).

**Figure 2:**
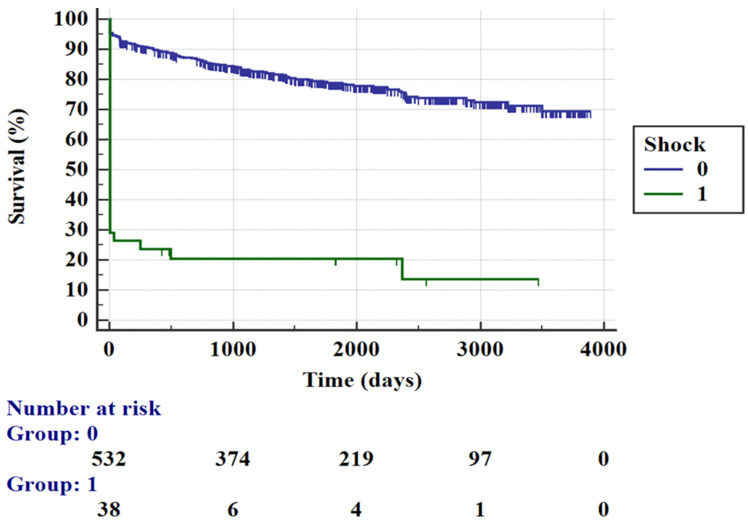
Kaplan–Meier curves for mortality rate according to the presence of cardiogenic shock at admission.

A total of 38 patients (6.5% of the whole population) presented with CS with, as expected, high in-hospital and long-term mortality rates, 68.4% and 41.7%, respectively (Figure 2).

In this subgroup, MVD was detected in 21 patients (55.2%); in particular, 9 of them (23.7%) showed three-vessels disease. The mean LV-EF was 28.4 +/− 12.2%, and an IABP was placed in 21 patients (55.2%). Complete revascularization (CR) during index procedure was performed in 42.8% (*n* = 9) of the patients, and in this case the mortality rate also was extremely high; in fact, 44.4% of patients died during hospitalization, and 40% died during the follow-up period. The concomitant approach with IABP support and CR was applied in a total of six patients (15.8%), but we did not find any significant impact of IABP support and CR on in-hospital mortality. Finally, focusing on the high-risk subgroup of MVD (*n* = 245), which represents 42% of the entire population, we divided patients into two groups according to the PCI strategy: CR *n* = 47 vs. culprit only (CO) revascularization *n* = 198. Patients’ baseline clinical and procedural characteristics are listed in Table 3.

**Figure 3:**
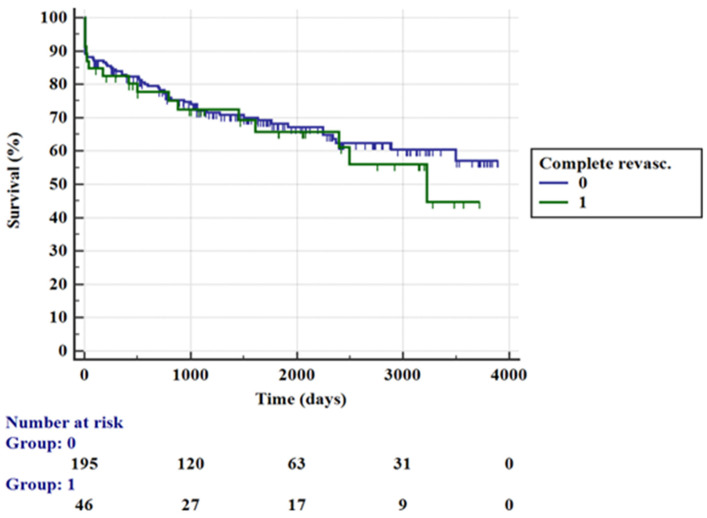
Kaplan–Meier curves for mortality rate in multivessel coronary artery disease group according to PCI strategy.

Patients treated with CR were more often in CS at admission (19.1% vs. 6.1%, *p* = 0.008) and showed a high rate of two-vessel disease (80.9% vs. 51.5%), while a lower incidence of patients presented with three-vessel disease (19.1% vs. 48.5%) at coronary angiography (*p* = 0.001). No other significant differences were found when comparing the two groups. In-hospital mortality was 18.6% and was not significantly influenced by the PCI strategy (8.5% vs. 10.1%, *p* = 0.99). Similar results were found regarding long-term mortality (31% vs. 24.6%, *p* = 0.43) and 1-year mortality (10% vs. 7.8%, *p* = 0.65). The absence of association between PCI strategy and mortality rate was confirmed by Cox regression analysis (HR [95% CI] = 1.31 [0.71–2.44], *p* = 0.39) and represented in terms of survival among the two groups at the Kaplan–Meier curve (Figure 3).

The main predictors for mortality were age (HR 1.12, *p* < 0.001), lower LV-EF (HR 0.97, *p* = 0.02) and Killip class ≥3 at admission (HR 2.29, *p* = 0.04) (Table 4). CS at admission was found in 21 patients with MVD, and a PCI strategy with CR was performed in the 42.9% of cases instead of the 17% for hemodynamically stable patients (*p* = 0.008). Regarding in-hospital and 1-year mortality, the main predictors were the same as the general population of anterior STEMI. In particular, LV-EF (OR 0.91, *p* = 0.01), baseline eGFR (OR 0.97, *p* = 0.04), female gender (OR 14.06, *p* = 0.005) and shock at admission (OR 133.3, *p* < 0.001) for in-hospital mortality and age (OR 1.15, *p* < 0.001) and Killip class ≥ 3 (OR 4.17, *p* = 0.04) for 1-year mortality (Table 4).

## 4. Discussion

In this study we focused our attention on patients with anterior STEMI that represent a population at very high risk of in-hospital and follow-up complications. In the total cohort of our population, the all-cause mortality percentage was significantly high and in line with the results of previous studies [19] (8.6% for in-hospital time and 18.8% for long-term follow-up). In particular, the in-hospital death rate was significantly higher for female patients with signs of CS at admission, severe left ventricular systolic dysfunction and lower eGFR. The main predictors of 1-year mortality were age and Killip class ≥ 3 at admission, while during long-term follow up, age, CAD extension and lower LV-EF were associated with a higher mortality rate. This is an interestingly result since we can appreciate that lower LV-EF correlates both with in-hospital and long-term mortality, certainly through two different pathophysiological mechanisms. Concerning in-hospital mortality, severe left ventricular dysfunction in A-STEMI frequently coincides with the presence of CS at admission and therefore, as expected, confirms a stronger association between these two elements [20]. In addition to AMI, the acute decline in LV function also manifests as a result of both transient stunned and hibernating myocardium that are potentially reversible once blood flow is restored. LV-EF was also shown to be a consistent in-hospital predictor of mortality regardless of the state of hemodynamic instability [21]. On the other hand, poor cardiac function influences long-term mortality, as it represents ventricular remodeling and chamber dilatation with a secondary volume-overload hypertrophy and is therefore a marker of predisposition to chronic heart failure (HF) [22]. The recognition of these precursors is important because they are related to poor outcomes, and starting treatment during the primary phase may reduce mortality in patients with asymptomatic systolic LV dysfunction [23]. As reported in previous studies, up to 65% of patients presenting with STEMI had MVD with concomitant obstructive non-IRA stenosis. Our results confirm this trend, showing multivessel obstructive disease in almost half of the cases. As already emphasized, the extent of coronary disease negatively influences long-term outcome. Based on these data, we focused our attention on the two higher risk subgroup that are represented by those with CS and those with MVD. The first group comprised 6.5% of the entire population, and this percentage is similar with the incidence of the CS complicating AMI in different European countries [24]. In-hospital mortality for those patients was very high, as exemplified by the Kaplan–Meier curve in Figure 2. Hemodynamic unstable patients were more often treated with complete one-stage revascularization according to current literature recommendations. Within this group we noted no significant differences in in-hospital mortality with the use of IABP support associated with CR strategy as previously demonstrated by several studies [25,26]. These results are also consistent with the current evidence, which does not recommend the routine use of IABP in CS patients. Anyway, due to the small sample size of this high-risk subgroup of patients, the statistical power of this analysis was limited, although it is in line with current literature data. On the other hand, focusing on patients with MVD, they were divided in two groups according to the PCI strategy (one-stage multivessel PCI vs. culprit-only PCI) and also in this case, the results showed a lack of association between PCI strategy and mortality rate. Predictors of mortality were basically the same as those found in the general population. Current literature data [6,7,8,12] confirm the benefit of CR in patients with STEMI and MVD; however, a strict recommendation regarding the best timing is lacking, except for those patients with hemodynamic instability. Our results are coherent with these studies, which do not demonstrate any benefit of the one-stage multivessel PCI approach in terms of its impact on the mortality rate. In this challenging scenario, an index multivessel approach does not seem to be associated with a better outcome in stable patients, and there is not currently enough information to support any recommendations regarding the optimal management of STEMI and complex MVD.

### Study Limitations

This is a retrospective monocentric study with a relatively small sample size, especially regarding MI complicated by CS. This characteristic, of course, limited the feasibility and the strength of additional subgroups analysis. Furthermore, it was difficult to explore full clinical data and biochemistry, in particular concerning the first years of analysis when only a few data were available for all patients.

## 5. Conclusions

Our study confirmed the high mortality rate of A-STEMI patients both at the short- and long-term follow up, especially in case of AMI complicated by CS. LV-EF is a powerful predictor of a poor outcome and should therefore be a part of the routine evaluation both for in-hospital risk stratification and also for a close long-term follow-up. In MVD, complete one-stage revascularization during primary PCI did not show any advantage in terms of reduction in in-hospital, short-term and long-term mortality when compared to culprit-only PCI strategy. Therefore, to date, one-stage CR should be reserved only for AMI patients presenting with CS. Further analysis is needed to investigate alternative approaches to non-culprit stenosis, such as functionally guided PCI during the index procedure.

## Figures and Tables

**Table 1:** Clinical and procedural characteristics.

Baseline Clinical Characteristics				
	Whole Population (*n* = 584)	Survival (*n* = 423)	Non-Survival (*n* = 161)	*p* Value
Age (M-SD)	66.2 +/− 13.6	62.5 +/− 12.2	77.5 +/− 10.8	<0.001
Male gender (%)	70.7	75.6	56.8	<0.001
Anemia (%)	20.3	18.2	26.4	<0.001
Renal function				<0.001
eGFR > 90 mL/min/1.73 mq (%)	38.8	45.5	18.2	
90 < eGFR < 60 mL/min/1.73 mq (%)	38.4	40.8	33.6	
60 < eGFR < 30 mL/min/1.73 mq (%)	17.7	11.8	35	
30 < eGFR< 15 mL/min/1.73 mq (%)	2.3	1	6.3	
eGFR < 15 mL/min/1.73 mq (%)	2.6	1	7	
Diabetes (%)	18.2	16.4	23	Ns
Hypertension (%)	49.7	46.7	58.8	0.04
Family history of CAD (%)	13.7	16.8	4.1	<0.001
Active smoke (%)	38	44.1	21.2	<0.001
Previous smoke (%)	2.4	2.8	1.4	<0.001
Dyslipidemia (%)	31.2	32.2	27	Ns
Overweight (%)	10.6	11.6	6.8	Ns
Ejection fraction (M-SD)	45.1 +/− 11	46.9 +/− 9.7	38.7 +/− 12.6	<0.001
History of CAD (%)	5.1	4.7	6.1	Ns
Killip class (%)				<0.001
Killip class 1	83.4	91	60.8	
Killip class 2	5.1	4.7	6.8	
Killip class 3	5	2.6	11.5	
Cardiogenic shock at presentation (%)	6.5	1.7	20.9	
Cardiac arrest (%)	3.3	1.7	7.4	<0.001
Haemoglobin (g/dL) (M-SD)	13.9 +/− 2	14.3 +/− 1.7	12.8 +/− 2.1	<0.001
Serum Creatinine (mg/dL) (M-SD)	1.08 +/− 0.81	0.97 +/− 0.6	1.37 +/− 1.08	<0.001
eGFR (mL/min/m^2^) (M-SD)	78.2 +/− 25.7	83.9 +/− 22.1	65.5 +/− 28.1	<0.001
Procedural characteristics				
CAD extension				
One vessel disease (%)	46.6	56.7	39.1	<0.001
Two vessels disease (%)	24	22.1	28.7	Ns
Three vessels disease (%)	18	11.6	26.3	<0.001
Left anterior descending (%)	82.9	88.2	85.6	Ns
Left main (%)	3.1	0.2	10.5	<0.001
Diagonal branch (%)	1	1.4	-	<0.001
Non obstructive coronary arteries (%)	11.4	9.6	3.9	<0.001
Femoral access (%)	63	52.6	74.3	<0.001
Radial access (%)	37	47.4	25.7	<0.001
Intra aortic balloon pump (%)	4.8	1.9	13.5	<0.001
Manual thrombectomy (%)	31.3	34.1	25	0.05
Contrast volume (mL) (M-SD)	172 +/− 26	165 +/− 24	174 +/− 28	Ns

M-SD: mean-standard deviation; eGFR:estimated glomerular filtration rate ; CAD: coronary artery disease.

**Table 2:** Main independent predictors for short- and long-term mortality.

Predictors for Long Term Mortality (Cox Proportional Hazards Regression)	
Age	HR [95% CI] = 1.12 [1.08–1.15] *p* < 0.001
LV-EF	HR [95% CI] = 0.97 [0.95–0.99] *p* = 0.02
Killip class at admission ≥ 3	HR [95% CI] = 2.29 [1.01–5.19] *p* = 0.04
* female gender, hypertension, diabetes, eGFR, Killip class and IABP	
Predictors for 1-year mortality (logistic regression)	
Age	OR [95% CI] = 1.15 [1.07–1.25] *p* < 0.001
Killip class at admission ≥ 3	OR [95% CI] = 4.17 [1.02–17.11] *p* = 0.04
* female gender, hypertension, diabetes, eGFR, CA, LV-EF, IABP, CS and anemia	
Predictors for in-hospital mortality (logistic regression)	
LV-EF	OR [95% CI] = 0.91 [0.85–0.98] *p* = 0.01
Baseline eGFR	OR [95% CI] = 0.97 [0.94–0.99] *p* = 0.04
Female gender	OR [95% CI] = 14.06 [2.24–88.10] *p* = 0.005
Cardiogenic shock at admission	OR [95% CI] = 133.3 [9.08–1956.44] *p* < 0.001
* age, Killip class, diabetes, IABP and anemia	

LV-EF: left ventricular ejection fraction; CA: cardiac arrest; IABP: intra aortic balloon pump; CS: cardiogenic shock; * non significative variables excluded in the model.

**Table 3:** Clinical and procedural characteristics of patients with MVD.

Variable	Revascularization		
**Clinical characteristics**	Complete revascularization (*n* = 47)	Culprit only (*n* = 198)	*p*-value
Age (M-SD)	69.8 +/− 14.2	67.6 +/− 12.9	0.32
Male sex (%)	68.1	78.3	0.18
Hypertension (%)	63.8	55.6	0.33
Smokers (%)			0.06
Active smokers (%)	25.5	40.6	
Previous smokers (%)	2.1	3	
Hypercolesterolemia (%)	36.2	30.8	0.49
Family history of CAD (%)	14.9	8.6	0.27
Overweight (%)	12.8	10.6	0.61
Diabetes (%)	27.7	21.7	0.44
History of CAD (%)	8.5	5.1	0.32
Cardiogenic shock (%)	19.1	6.1	0.008
Renal function			0.20
eGFR > 90 mL/min/1.73 mq) (%)	28.3	34.5	
Mild reduction (90 < eGFR < 60 mL/min/1.73 mq) (%)	34.8	38.7	
Moderate reduction (60 < eGFR < 30 mL/min/1.73 mq) (%)	28.3	20.6	
Severe reduction (30 < eGFR < 15 mL/min/1.73 mq) (%)	4.3	3.6	
End-stage renal disease (eGFR < 15 mL/min/1.73 mq) (%)	4.3	2.6	
Ejection fraction (M-SD)	42.6 +/− 13.5	44.2 +/− 10.7	0.40
Killip class (%)			
Killip class 1	61.7	78.2	0.01
Killip class 2	12.8	6.6	
Killip class 3	6.4	9.1	
Killip class 4	19.1	6.1	
Anemia (%)	21.7	24.7	0.85
Haemoglobin (g/dL) (M-SD)	13.8 +/− 2.5	13.8 +/− 2.0	0.98
Serum Creatinine (mg/dL) (M-SD)	1.25 +/− 0.96	1.14 +/− 0.87	0.44
eGFR (mL/min/m^2^) (M-SD)	68.7 +/− 27.57	75.4 +/− 25.8	0.12
**Procedural characteristics**			
CAD extension (%)			<0.001
Two vessels disease	80.9	51.5	
Three vessels disease	19.1	48.5	
Femoral access (%)	68.1	63.7	0.24
Radial access (%)	31.9	36.4	
Intra aortic balloon pump (%)	12.8	6.6	0.22
Manual thrombectomy (%)	34.8	23.4	0.13
In-hospital mortality (%)	8.5	10.1	0.99
Long term mortality (%)	31	24.6	0.43
1 year mortality (%)	10	7.8	0.65
Follow up days (M-SD)	1635.1 +/− 1190	1698.3 +/− 1127.4	0.75

**Table 4:** Main independent predictors for short- and long-term mortality in patients with MVD.

Predictors for Long Term Mortality (Cox Proportional Hazards Regression)	
Age	HR [95% CI] = 1.12 [1.09–1.14] *p* < 0.001
CAD extension	HR [95% CI] = 1.41 [1.12–1.79] *p* = 0.003
LV-EF	HR [95% CI] = 0.96 [0.94–0.98] *p* < 0.001
* female gender, diabetes, eGFR, anemia, CS and CR	
Predictors for 1-year mortality (logistic regression)	
Age	OR [95% CI] = 1.15 [1.07–1.24] *p* = 0.003
Killip class at admission ≥ 3	OR [95% CI] = 4.17 [1.02–17.11] *p* = 0.04
* LVEF, hypertension, CS, female gender, diabetes, eGFR, CR and anemia	
Predictors for in-hospital mortality (logistic regression)	
LV-EF	OR [95% CI] = 0.93 [0.87–0.98] *p* = 0.017
Baseline eGFR	OR [95% CI] = 0.97 [0.94–0.99] *p* = 0.03
Female gender	OR [95% CI] = 9.06 [1.66–49.37] *p* = 0.01
Cardiogenic shock at admission	OR [95% CI] = 29.56 [4.22–206.91] *p* < 0.001
* age, Killip class, diabetes, IABP and anemia	

* non significative variables included in the model.
